# Mathematical Modelling of Cell-Fate Decision in Response to Death Receptor Engagement

**DOI:** 10.1371/journal.pcbi.1000702

**Published:** 2010-03-05

**Authors:** Laurence Calzone, Laurent Tournier, Simon Fourquet, Denis Thieffry, Boris Zhivotovsky, Emmanuel Barillot, Andrei Zinovyev

**Affiliations:** 1 Institut Curie, Paris, France; 2 Ecole des Mines ParisTech, Paris, France; 3 INSERM U900, Paris, France; 4 TAGC – INSERM U928 & Université de la Méditerranée, Marseille, France; 5 CONTRAINTES Project, INRIA Paris-Rocquencourt, France; 6 Karolinska Institutet, Stockholm, Sweden; UT Southwestern Medical Center, United States of America

## Abstract

Cytokines such as TNF and FASL can trigger death or survival depending on cell lines and cellular conditions. The mechanistic details of how a cell chooses among these cell fates are still unclear. The understanding of these processes is important since they are altered in many diseases, including cancer and AIDS. Using a discrete modelling formalism, we present a mathematical model of cell fate decision recapitulating and integrating the most consistent facts extracted from the literature. This model provides a generic high-level view of the interplays between NFκB pro-survival pathway, RIP1-dependent necrosis, and the apoptosis pathway in response to death receptor-mediated signals. Wild type simulations demonstrate robust segregation of cellular responses to receptor engagement. Model simulations recapitulate documented phenotypes of protein knockdowns and enable the prediction of the effects of novel knockdowns. *In silico* experiments simulate the outcomes following ligand removal at different stages, and suggest experimental approaches to further validate and specialise the model for particular cell types. We also propose a reduced conceptual model implementing the logic of the decision process. This analysis gives specific predictions regarding cross-talks between the three pathways, as well as the transient role of RIP1 protein in necrosis, and confirms the phenotypes of novel perturbations. Our wild type and mutant simulations provide novel insights to restore apoptosis in defective cells. The model analysis expands our understanding of how cell fate decision is made. Moreover, our current model can be used to assess contradictory or controversial data from the literature. Ultimately, it constitutes a valuable reasoning tool to delineate novel experiments.

## Author Summary

Activation of death receptors (TNFR and Fas) can trigger either survival or cell death according to the cell type and the cellular conditions. In other words, the same signal can have antagonist responses. On one hand, the cell can survive by activating the NFκB signalling pathway. On the other hand, it can die by apoptosis or necrosis. Apoptosis is a suicide mechanism, i.e., an orchestrated way to disrupt cellular components and pack them into specialized vesicles that can be easily removed from the environment, whereas necrosis is a type of death that involves release of intracellular components in the surrounding tissues, possibly causing inflammatory response and severe injury. We, biologists and theoreticians, have recapitulated and integrated known biological data from the literature into an influence diagram describing the molecular events leading to each possible outcome. The diagram has been translated into a dynamical Boolean model. Simulations of wild type, mutant cells and drug treatments qualitatively match current data, and predict several novel mutant phenotypes, along with general characteristics of the cell fate decision mechanism: transient activation of some key proteins in necrosis, mutual inhibitory cross-talks between the three pathways. Our model can further be used to assess contradictory data and address specific biological questions through *in silico* experiments.

## Introduction

Engagement of TNF or FAS receptors can trigger cell death by apoptosis or necrosis, or yet lead to the activation of pro-survival signalling pathway(s), such as NFκB. Apoptosis represents a tightly controlled mechanism of cell death that is triggered by internal or external death signals or stresses. This mechanism involves a sequence of biochemical and morphological changes resulting in the vacuolisation of cellular content, followed by its phagocyte-mediated elimination. This physiological process regulates cell homeostasis, development, and clearance of damaged, virus-infected or cancer cells. In contrast, pathological necrosis results in plasma membrane disruption and release of intracellular content that can trigger inflammation in the neighbouring tissues. Long seen as an accidental cell death, necrosis also appears regulated and possibly involved in the clearance of virus-infected or cancer cells that escaped apoptosis [1].

Dynamical modelling of the regulatory network controlling apoptosis, non-apoptotic cell death and survival pathways could help identify how and under which conditions the cell chooses between different types of cellular deaths or survival. Moreover, modelling could suggest ways to re-establish the apoptotic death when it is altered, or yet to trigger necrosis in apoptosis-resistant cells. The decision process involves several signalling pathways, as well as multiple positive and negative regulatory circuits. Mathematical modelling provides a rigorous integrative approach to understand and analyse the dynamical behaviours of such complex systems.

Published models of cell death control usually focus on one death pathway only, such as the apoptotic extrinsic or intrinsic pathways [2],[3],[4]. A few studies integrate both pathways [5], some show that the concentration of specific components contribute to the decision between death and survival [6],[7] while other studies investigate the balance between proliferation, survival or apoptosis in specific cell types along with the role of key components in these pathways [8], but no mathematical models including necrosis are available yet. Moreover, we still lack models properly demonstrating how cellular conditions determine the choice between necrosis, apoptosis and survival, and how and to what extent conversions are allowed between these fates.

**Figure 1:**
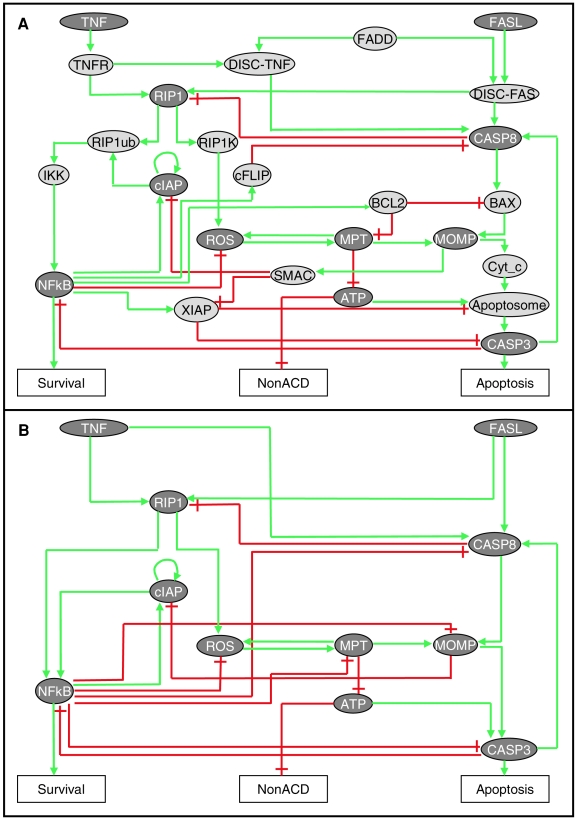
Regulatory networks of cell-fate decision. (A) Master model: the molecular interactions between the main components intervening in the three pathways are described as an influence graph leading to the three cell fates: survival, non-apoptotic cell death and apoptosis. Dashed lines denote the pathway borders. (B) The corresponding reduced model.

Our study aims at identifying determinants of this cell fate decision process. The three main phenotypes considered are *apoptosis*, *non-apoptotic cell death* (which mainly covers *necrosis*) and *survival*. Although the pathways leading to these three phenotypes are highly intertwined, we first describe them separately hereafter, concentrating on the players we chose to include in each pathway. Summarised in Figure 1A, this description does not intend to be exhaustive, but rather aims at covering the most established processes participating in cell fate decision.

### Caspase-dependent apoptotic cell death

Only the apoptotic caspase-dependent pathway downstream of FAS and TNF receptors is considered here. Upon engagement by their ligands and in the presence of FADD (FAS-Associated protein with Death Domain), a specific Death Inducible Signalling Complex (DISC-FAS or DISC-TNF in Figure 1) forms and recruits pro-caspase-8. This leads to the cleavage and activation of caspase-8 (CASP8). In the so-called type II cells, CASP8 triggers the intrinsic or mitochondria-dependent apoptotic pathway, which also responds to DNA damage directly through the p53-mediated chain of events (not detailed here). CASP8 cleaves the BH3-only protein BID (not explicitly included in the diagram), which can then translocate to the mitochondria outer membrane. There, BID competes with anti-apoptotic BH3 family members such as BCL2 for interaction with the proteins BAX or BAK (BAX will stand here for both BAX and BAK). Consequently, oligomerisation of BAX results in mitochondrial outer membrane permeabilisation (MOMP) and the release of pro-apoptotic factors. Once released to the cytosol, cytochrome c (Cyt_c) interacts with APAF1, recruiting pro-caspase-9. In presence of dATP, this enables the assembly of the apoptosome complex (referred to as ‘Apoptosome’ in Figure 1A, lumping APAF1 and pro-caspase-9), responsible for caspase-9 activation, followed by the proteolytic activation of pro-caspase-3 (CASP3) [9]. By cleavage of specific targets, the executioner caspases (CASP3 in the model) are responsible for major biochemical and morphological changes characteristic of apoptosis. SMAC/DIABLO (SMAC) is released during MOMP to the cytosol, where it is able to inactivate the caspase inhibitor XIAP [10]. CASP3 also participates in a positive circuit by inducing the activation of CASP8 [11],[12]. In type I cells, CASP8 directly cleaves and activates executioner caspases such as CASP3 (not described).

### Non-apoptotic cell death (NonACD)

Here, we consider mainly a mode of cell death with morphological features of necrosis, which occurs when apoptosis is impeded in cells treated with cytokines [13] or in some specific cell lines such as L929 cells when exposed to TNF [14]. In primary T cells, if caspases are inhibited, activation of TNFR or FAS causes necrosis via a pathway that requires the protein RIP1 and its kinase activity (RIP1K) [13]. This RIP1-dependent cytokine-induced necrotic death defines necroptosis [15],[16]. A genetic screen recently identified other genes necessary for this type of cell death [17]. However, a precise description of this pathway is still lacking. Reactive oxygen species (ROS) were proposed to be involved downstream of RIP1 [18]. ROS are also thought to play a key role in the control of mitochondria permeability transition (MPT), since they are produced by damaged mitochondria and can oxidize mitochondrial components, thus favouring MPT [19],[20],[21]. Furthermore, the role of mitochondria in necrosis is highlighted through the involvement of MPT, which causes a fatal drop in ATP level and leads to necrotic death. Indeed, MPT results from the inhibition of ATP/ADP exchange at the level of mitochondrial membranes, or from the inhibition of oxidative phosphorylation decreasing cellular ATP level and causing energy failure [21],[22]. Although there is evidence that necrosis is also triggered by TNF- and FAS-independent pathways, these are not yet considered in this study. These pathways include, for example, calpain-mediated cleavage of AIF followed by its nuclear translocation [23],[24], or PARP-1-mediated NAD^+^ depletion [24],[25].

### Survival pathway

NFκB represents a family of transcription factors that play a central role in inflammation, immune response to infections and cancer development [26]. The ubiquitination of RIP1 at lysine 63 by cIAP leads to the activation of IKK and ultimately that of NFκB [27]. In different cell types, especially in tumour cell lines, activation of NFκB inhibits TNF-induced cell death [28]. This effect is mediated by NFκB target genes: cFLIP inhibits recruitment of CASP8 by FADD [29]; anti-apoptotic BCL2 family members inhibit MOMP and MPT [30],[31],[32]; XIAP acts as a caspase inhibitor [33]; and ferritin heavy chain [34] or mitochondrial SOD2 [35] decrease ROS levels (these mechanisms are represented in Figure 1A by a direct inhibitory arrow from NFκB to ROS). For the sake of simplicity, other NFκB target genes that are known to inhibit TNF-induced apoptosis are provisionally omitted in the model (e.g., A20; cf. [36],[37]).

Our goal here is to provide a simplified but yet rigorous model of the mechanisms underlying cell fate selection in response to the engagement of FAS and TNF receptors. We have proceeded in several steps. First, we have assembled a regulatory network covering the main experimental data. Species and interactions were selected on the basis of an extensive literature search and integrated in the form of a diagram or “regulatory graph”. This diagram is then translated into a dynamical model. Our analysis initially focuses on the determination of the asymptotic properties of the system for different conditions, which correspond to the possible phenotypes that the model can account for. Next, we analyse the different trajectories leading to each phenotype in the wild type and mutant situations. As quantitative data are still largely lacking for this system, we use a qualitative logical formalism and its implementation in the *GINsim* software [38]. As we shall see, proper model analysis can assess where and when cell fate decisions are made, provide novel insight concerning the general structure of the network, in particular concerning the occurrence of cross-talks between pathways, and predict novel mutant phenotypes and component activity patterns.

## Results

The information gathered in the literature has been integrated into a regulatory graph (Figure 1A). Our selection of molecular players (nodes of the graph) is based on our current understanding of the molecular mechanisms of cell fate decision. Documented positive or negative effects among pairs of components are represented by signed arcs (Figure 1). Each node or arc is annotated and associated with bibliographical references in the model file, as well as in the accompanying documentation (cf. supplementary Text S1).

Our current model encompasses three main pathways (Figure 1A): the activation of a caspase-dependent apoptosis pathway, the RIP1-kinase-dependent pathway leading to necrosis, and the activation of the transcription factor NFκB with pro-survival effects. Other pathways involved in cell death, such as growth factor receptors or other RTK (receptor tyrosine kinase), TLR (Toll-like receptor), and MAPK signalling pathways have been provisionally left out.

We defined specific “markers” or “read-outs” of the three cell fates. When caspase-3 is activated, the cells are considered to be apoptotic; when MPT occurs and the level of ATP drops dramatically, the cells enter non-apoptotic cell death; finally, when NFκB is activated, we consider that cells survive. Active survival is thus monitored here by the activation of NFκB pathway, in accordance with many studies, as opposed to passive survival, which occurs when no death signals are engaged. In reality, other pathways can interact downstream of NFκB activation, which can reinforce or shut off survival. For now, passive survival will be referred to as the ‘naïve’ state, i.e. the stable states with none of the three pathways activated.

### Cross-talks between the three pathways

As mentioned before, the pathways are highly intertwined (Figure 1A). For instance, the survival pathway interacts with the apoptotic pathway at different points: cFLIP inhibits CASP8; BCL2 blocks mitochondria pore opening through inhibition of BAX (and BAK, implicitly represented in our model); and XIAP blocks the activity of both CASP9 in the apoptosome and CASP3. Conversely, the apoptotic pathway negatively regulates NFκB activity through the CASP8-mediated cleavage and inactivation of RIP1 upstream of NFκB. Because RIP1 operates upstream of the necrotic pathway, this regulation also impacts necrosis. Moreover, for the apoptosome to form, dATP (or/and ATP) is (/are) needed. Consequently, in our model, when necrosis occurs, ATP production drops, terminating apoptosis. Regarding the influence of the survival pathway on the necrotic one, NFκB tentatively stimulates the production of anti-oxidants that shuts off ROS level. Both the necrotic and the apoptotic pathways are able to interact with the survival pathway through the action of cIAP1/2, referred to as cIAP in our model. More precisely, cIAP1 and 2 are E3-ubiquitin ligases that target RIP1 for K63-linked polyubiquitination. They are essential intermediates in the activation of NFκB downstream of TNF receptor [27]. Some synthetic molecules that mimic the N-terminal of SMAC IAP-interacting motif have been shown to induce cIAP1/2 auto-ubiquitination and subsequent proteasomal degradation, thus blocking TNF-dependent NFκB activation [39],[40]. Tentatively, mitochondrial permeabilization in the apoptosis or necrosis pathways could block TNF-induced NFκB activation through the release of SMAC into the cytosol thereby causing the inhibition of c-IAP1/2. Initially, cIAP was not included in the model, which led to discrepancies between model simulations and published data. Indeed, in *FADD* or *CASP8* deletion mutants, our preliminary model predicted only survival (not shown), whereas both necrotic and survival phenotypes were observed in experiments in the presence of TNF or FAS [41],[42],[43]. The consideration of the path MOMP⇒SMAC = |cIAP⇒NFκB enabled us to eliminate the discrepancies, both necrotic and survival phenotypes were then obtained in the simulations, although it does not preclude other mechanisms.

### Dynamical logical model of cell fate decision

To transform the static map shown in Figure 1A into a dynamical model accounting for the different scenarios or set of events leading to one of the three phenotypes, we have to define proper dynamical rules. Since there is little reliable quantitative information on reaction kinetics and cellular conditions leading to one or another phenotype, these rules must be sufficiently flexible to cover all possible scenarios following death receptor activation.

The nodes encompassed in the map represent different things: simple biochemical components (receptors, ligands, proteins or metabolites): TNF, FASL, TNFR, FADD, FLIP, CASP8, RIP1, IKK, NFκB, cIAP, BCL2, BAX, Cyt_c, SMAC, ROS, XIAP, CASP3, ATP); specific modified forms of proteins: RIP1K (active RIP1 kinase), RIP1ub (K63-ubiquitinated RIP1); complexes of proteins: DISC-TNF (corresponding to TRADD, TRAF2, FADD, proCASP8), DISC-FAS (corresponding to FAS, FADD, proCASP8), apoptosome; cellular processes: MPT (Mitochondrial Permeability Transition) and MOMP (Mitochondria Outer Membrane Permeabilisation).

A Boolean variable is associated with each of these nodes, which can take only two logical values: “0” (false), denoting the absence or inactivity of the corresponding component, and “1” (true), denoting its active state.

Furthermore, a logical rule (or function) is assigned to each node, defining how the different inputs (incoming arrows) combine to control its level of activation. For example, CASP8 can be activated (its value is set to “1”) by DISC-TNF or DISC-FAS, but only in the absence of cFLIP protein. This can be encoded into a logical rule as follows: (DISC-TNF OR DISC-FAS) AND NOT cFLIP. Several nodes correspond to simple inputs (TNF, FASL and FADD). Their initial values are kept fixed during most simulations.

On the basis of the regulatory graph and the associated logical rules, we then proceeded with the exploration of the dynamical properties of our model. We first focused on the identification of all stable states and on their biological interpretation. Then, we investigated the reachability of these stable states for different initial conditions, for both wild type and mutant cases. Details on the computational methods used are provided in the Methods section and in the supplementary Text S1. The logical model has been filed in the BioModels database with the reference MODEL0912180000.

### Identification of stable states

**Figure 2:**
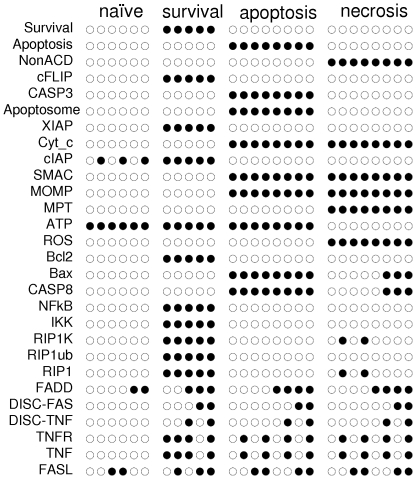
Stable states of the master model. values 0 and 1 are represented by empty and full circles, respectively.To compare these stable states to those of the simplified model, the values of FADD = 0 need to be deleted since FADD is not explicitly presented in the reduced model. The first six rows (with NonACD = 0, Apoptosis = 0, Survival = 0) correspond to the “naïve” state. The following five rows (with NonACD = 0, Apoptosis = 0, Survival = 1) correspond to ‘survival’. The following eight rows (with NonACD = 0, Apoptosis = 1, Survival = 0) correspond to ‘apoptosis’. The last eight rows (with NonACD = 1, Apoptosis = 0, Survival = 0) correspond to ‘necrosis’.

Analysis of the cell fate decision model (Figure 1A) led to the identification of the 27 stable states showed in Figure 2. These stable states are the sole attractors of the system under the asynchronous assumption (see Methods). They thus represent all possible cellular asymptotical states. In other words, whatever the initial conditions, a wild type cell will end up in one of these states if we wait long enough. A closer look reveals that several stable states correspond to each cellular fate, with few differing (minor) component values.

This consideration led us to address the following questions: (i) Does a cluster structure exist in the distribution of internal stable states of the network? (ii) If so, in these clusters, could the corresponding states be interpreted as slightly different realisations of the same cellular phenotype? (iii) What would be the characteristic signature of each cluster (conserved values of variables inside each cluster)? (iv) What is the number of independent variables defining the internal stable states of the network?

**Figure 3:**
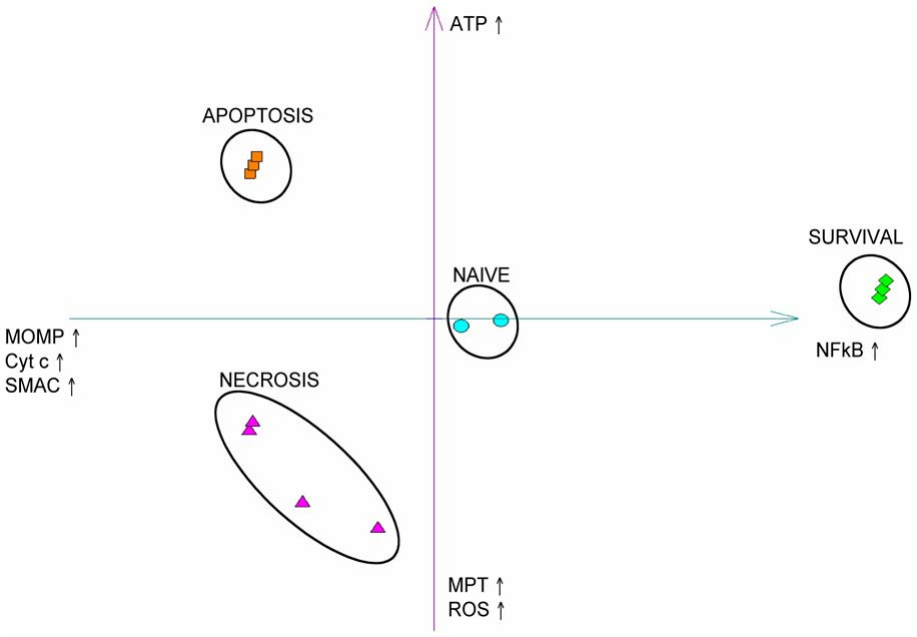
Projection of the internal stable state values onto the first two principal components. Four clusters are formed: the ‘naïve’ cluster (round light blue circles) at the center; the survival cluster (green rhombs) characterised by high level of NFκB and related (see Table S1 for details); the apoptosis cluster (orange squares) characterised by high levels of MOMP, Cyt_c, SMAC and ATP; and the non-apoptotic cell death - or necrosis - cluster (purple triangles) with high levels of MPT, ROS, MOMP, Cyt_c and SMAC. In the latter three clusters, there are three subgroups of stable states which correspond to the different inputs of the system: top stable states correspond to high TNF and FAS signals, middle stable states have either one or the other signal while the lower ones have no inputs. As for the naive cluster, the two sets of stable states differ by their cIAP value. Inputs and outputs are not included in the stable state binary vector.

Standard statistical methods and clustering algorithms are applied to group stable states. Figure 3 displays a projection of the internal (without inputs and outputs) stable values into the 2D space defined by the first two principal components of the corresponding distribution. The first two principal components explain 52% and 20% of the total variation, respectively (Table S1). The first principal component can be associated with the activity of NFκB pathway, while the second is determined mainly by ATP and MPT status. These factors do appear to determine the principal (independent) degrees of freedom for the internal state of the network. A typical trajectory starting from any set of initial conditions will thus quickly converge to the region under the influence of these three components.

The 2-D graph (Figure 3) reveals a striking separation of the stable states into 4 clusters: one cluster (blue circles) devoid of significant activity, which we call the ‘naïve’ state; one cluster (green rhombs) corresponding to survival, with NFκB pathway activated; and two clusters corresponding to the two different modalities of cell death, apoptosis (orange squares) and necrosis (purple triangles). K-means clustering using Euclidean and L1 distance perfectly reproduces these groupings, demonstrating that the compact groups easily distinguishable on the PCA plot indeed represent well-separated clusters in the original multidimensional space.

Some interesting conclusions and predictions can be drawn just by looking at the values of each component in each phenotypical group. For instance, in the necrotic (purple) stable states, when FADD is present (*i.e.* normal wild type conditions), RIP1 is always OFF and CASP8 ON, even though RIP1 is required and CASP8 is dispensable for necrosis to occur. This observation suggests a transient activation of RIP1 protein when switching on the necrotic pathway in response to death receptors. However, inactivation or cleavage of RIP1 is not *per se* a prerequisite for necrosis, nor is CASP8 activation. Indeed, for the mutant models in which CASP8 activation is impaired, such as *CASP8* or *FADD* deletion, there exist necrotic attractors with RIP1 = 1 (not shown). Our model thus predicts that TNF-induced necrosis could occur despite CASP8-mediated cleavage of RIP1. An attractive experimental model in which such a transient activation of RIP1 could be tested is the mouse fibrosarcoma cell line L929. Upon TNF exposure, these cells die by necrosis [44] and they have a functional CASP8 [45], which is cleaved during TNF-induced cell death [46].

Since RIP1 controls both the activation of NFκB and the level of ROS, the same transient behaviour could be expected for the survival phenotype. However, this is not observed with our model, as RIP1 = 1 in all survival (green) stable states. This can be explained by the regulatory circuit involving RIP1 and NFκB, which is not functional in necrosis. Indeed, when NFκB is active, it can mediate the synthesis of cFLIP, an inhibitor of CASP8, itself an inhibitor of RIP1. Moreover, RIP1 is part of the positive circuit that keeps NFκB ON. The model thus suggests that a sustained RIP1 activity is needed for survival. How could this hypothesis be experimentally assessed? If an experiment would reveal that RIP1 is only transiently activated upon death receptor activation, while NFκB remains activated, the model would be contradicted. In that case, one would need to look for other components capable to maintain NFκB active.

### Model reduction and dynamical analysis

The stable state analysis described above provides a first validation of the master model presented in Figure 1A. On this basis, we performed a more detailed analysis of the dynamics of the system. We investigated which cell fates (stable states) can be reached from specific initial conditions. Given a set of reachable stable states, can we say something about their relative “attractivity”?

To avoid the combinatorial explosion of the number of states to consider, we have reduced the number of components while preserving the relevant dynamical properties of the master model (Figure 1B). Details of how this reduction is performed are provided in the Methods section. The resulting network encompasses 11 components. The corresponding Boolean rules are listed in Table 1. The size of the transition graph (2^11^ = 2048) is now amenable to a detailed dynamical analysis. First, the set of attractors of this reduced model is identified: 13 attractors are obtained, which are all stable states matching those found for the master model when the input variable FADD = 1. Recall that, in the master model, both values of FADD were considered leading to 13 stable states with FADD = 1 and 14 stable states with FADD = 0. Using the theoretical results presented in [47] (mainly Theorem 1), we can conclude that the 13 stable states of Figure 2 are the only attractors of the master model when FADD = 1.

**Table 1:** Logical rules associated with the wild type reduced model.

Node	Logical update rule
TNF	*( INPUT NODE)*
FAS	*( INPUT NODE)*
RIP1′	NOT C8 AND (TNF OR FAS)
NFkB′	(cIAP AND RIP1) AND NOT C3
C8′	(TNF OR FAS OR C3) AND NOT NFkB
cIAP′	(NFkB OR cIAP) AND NOT MOMP
ATP′	NOT MPT
C3′	ATP AND MOMP AND NOT NFkB
ROS′	NOT NFkB AND (RIP1 OR MPT)
MOMP′	MPT OR (C8 AND NOT NFkB)
MPT′	ROS AND NOT NFkB

### Logical definition of “mutants”

Based on the reduced model defined in Figure 1B and Table 1, we derived 15 model variants representing biologically plausible perturbations. We will abusively use the term “mutant” to refer to these variants, even though they do not all technically correspond to mutations. For instance, the “z-VAD mutant” simulates the effect of caspase inhibitor z-VAD-fmk. Each mutant simulation consists in a local alteration of our reduced model, which can be qualitatively compared with results reported in the literature.

In the Boolean framework, such alterations amount to force the level of certain variables to zero in the case of a gene deletion, or to one in the case of a component over-expression. As we are using the reduced version of the master model, some perturbed components may be hidden by the reduction process. In such cases, we change the logical rules of their (possibly indirect) targets to take into account their effects. Table 2 lists the 15 variants of the model considered, along with the modified logical rules, the expected effects on the phenotypes according to the literature, and short descriptions of simulation results.

**Table 2:** Description of the different “mutant” versions of the reduced model.

Name	Modified rules	Expected phenotypes	Qualitative results
Anti-oxidant	ROS′ = (RIP1 OR MPT)	Prediction.	Suppression of NFκB anti-oxidant effect leads to no change in the decision process.
*APAF1* deletion	C3′ = 0	*APAF1* ^−/−^ mouse thymocytes are not impaired in FAS-mediated apoptosis ([71]).	Apoptosis disappears and replaced by the naïve state. Necrosis and survival are close to the wild type case situations.
*BAX* deletion	MOMP′ = MPT	*BAX* deletion blocks FAS or TNF+CHX - induced apoptosis in some cell lines, such as HCT116 [72].	*BAX* deletion prevents apoptosis.
*BCL2* over-expression	MOMP′ = MPT MPT′ = 0	FAS induces the activation of NFκB pathway [29].	As expected, the survival and naive attractors are preserved while both death pathways are inhibited.
*CASP8* deletion	C8′ = 0	*Caspase-8* deficient MEFs [41] or Jurkat cells [42] are resistant to FAS-mediated apoptotic cell death.	Apoptosis disappears. Compared to the wild type, a slight increase of necrosis is observed, while survival becomes the main cell fate.
constitutively activated CASP8	C8′ = 1	Prediction.	Over-expression of caspase-8 leads to a loss of NFκB activation.
*cFLIP* deletion	C8′ = TNF OR FAS OR C3	*cFLIP*−/− MEFs are highly sensitive to FASL and TNF [73].	The increase of apoptosis is effectively observed in the *cFLIP* mutant; furthermore survival can no longer be sustained.
*cIAP* deletion	cIAP′ = 0	NFκB activation in response to TNF is blocked [53].	NFκB activation is impaired, and only the apoptotic and necrotic attractors can be reached.
*FADD* deletion	C8′ = C3 AND NOT NFκB RIP1′ = NOT C8 AND TNF	*FADD* ^−/−^ mouse thymocytes are resistant to FAS mediated apoptosis [74]. *FADD* ^−/−^ MEFs are resistant to FASL and TNF [75]. In Jurkat cells treated with TNF+CHX, apoptosis is turned into necrosis [43].	FASL signalling is blocked and the ‘naïve’ attractor is the only reachable one. In response to TNF, apoptosis disappears.
*NFκB* deletion	NFkB′ = 0	TNF induces both apoptosis and necrosis in *NF-κB p65* ^−/−^ cells [76] or in IKKb^−/−^ fibroblasts [35].	This mutant shows a strong increase of necrosis (to be related with concomitant apoptosis/necrosis).
constitutively active NFκB	NFkB′ = 1	Prediction.	Both death pathways are shut down in this mutant.
*RIP1* deletion	RIP1′ = 0	*RIPK1* ^−/−^ MEFs are hypersensitivity to TNF, no TNF-induced NFκB activation, [77].	Both survival and necrosis states become unreachable. The effect of RIP1 silencing leads to a complete loss of the decision process (apoptosis becoming the only outcome).
*XIAP* deletion	C3′ = ATP AND MOMP	No effect on TNF-induced toxicity in *XIAP* ^−/−^ MEFs [78].	Behaviour similar to wild type.
z-VAD	C3′ = 0	FAS induced apoptosis is blocked, though cells can undergo death by necrosis [79]. FAS activates NFκB [80]. Induction of autophagic cell death observed [81].	The simulation of z-VAD mutant is similar to the silencing of caspase-8 (which implies that caspase-8 shut down in the model seems to have some priority over caspase-3 shutdown).
z-VAD+*RIP1* deletion	C3′ = 0 C8′ = 0 RIP1′ = 0	Upon TNF cells treated with z-VAD-fmk that are RIP-deficient cannot activate the NFκB pathway anymore and die by necrosis [13].	The conjugated effect of *RIP1* deletion and caspase inhibition impedes the system to trigger any of the three pathways (the ‘naïve’ state becomes the only possible outcome).

### Computation of reachable attractors

The references provided in Table 2 cover experiments performed on different cell types and with different experimental conditions. In contrast, our cell fate model represents mechanisms of cell fate decision in a generic cell, qualitatively recapitulating a wide variety of cellular contexts. Given a cellular system, its response to the activation of death receptors is determined by the logical rules. However, the generic model presented here considers equally all possible contexts and regulatory combinations. To evaluate the relative likelihood of having a particular response in a randomly chosen cellular system, we count the relative number of possible trajectories from the stimulated ‘naïve’ state to a given phenotype. This analysis gives an idea on what is possible or forbidden in a ‘generic’ cell.

**Figure 4:**
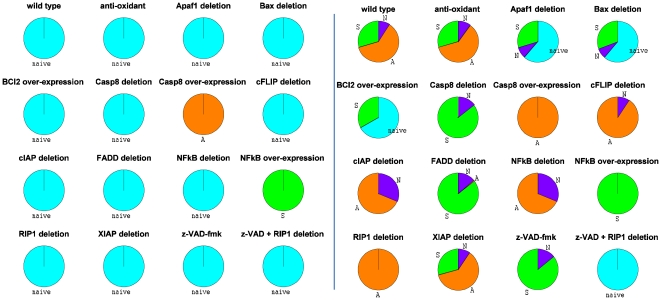
Reachability of phenotypes starting from “physiological” initial conditions. The colours correspond to the phenotypes as identified by the clustering algorithm (blue: “naïve” survival state; green: survival through NFκB pathway; orange: apoptosis; purple: necrosis). Left panel: TNF = FAS = 0, right panel: TNF = 1 and FAS = 0.

Using dedicated methods and software [48], the set of reachable stable states is calculated, starting from selected physiological initial conditions, for the wild type and mutant models. The physiological state is defined by fixing the variables ATP and cIAP to “1” and all the other ones to “0”. Different combinations of TNF and FASL are considered. The probability to reach each phenotype is computed as a fraction of the paths in the graph that link physiological initial conditions to each cell fate (Figure 4 for reduced model and Figure S4 for master model).

As expected, the absence of TNF and FASL can only lead to the ‘naïve’ state (except of course when caspase-8 or NFκB are over-expressed, for obvious reasons). This means that the inputs (TNF and FASL) are needed for the system to effectively trigger the decision process. This was expected since intracellular death signals are not yet taken into account in the model. When TNF = 1 (Figure 4, right panel), for the wild type system, we observe that three outcomes or phenotypes are reachable from the initial condition, with different probabilities: ∼10% for necrosis, ∼30% for active survival and ∼60% for apoptosis. Although these probabilities cannot be directly compared with experimental results, they become useful when comparing different variants of the model. For instance, an increase (or decrease) of a phenotype probability between the wild type and a particular mutant can be interpreted as a gain (or a loss) of effectiveness of the corresponding pathway in that mutant. Such qualitative observations can then be confronted with published experimental results, which are summarized in the last column of Table 2.

**Figure 5:**
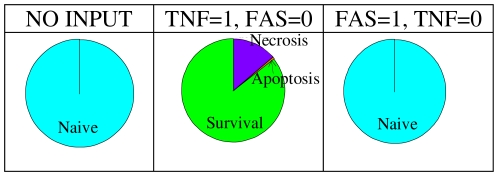
*FADD* mutant breaks the symmetry of TNF and FAS-induced pathways. Activation of death receptors in *FADD* deletion mutant leads to different cell fates depending on the values of TNF and FAS.

In most cases, activation of FASL and TNF lead to similar effects (not shown), except in the case of the *FADD* deletion mutant (Figure 5). As expected, this mutant cannot lead to cell death when FASL is ON. In contrast, necrosis is still possible in the presence of TNF. Interestingly, TNF-induced apoptosis is expected to be blocked [49] whereas the qualitative analysis shows that apoptosis is actually reachable in the model. Nevertheless, the probability of this phenotype is very low (around 0.61%), which means that very few trajectories may lead to apoptosis and it would thus be difficult to obtain the corresponding cellular context.

### Variation of the duration of receptor activation and its effects

**Figure 6:**
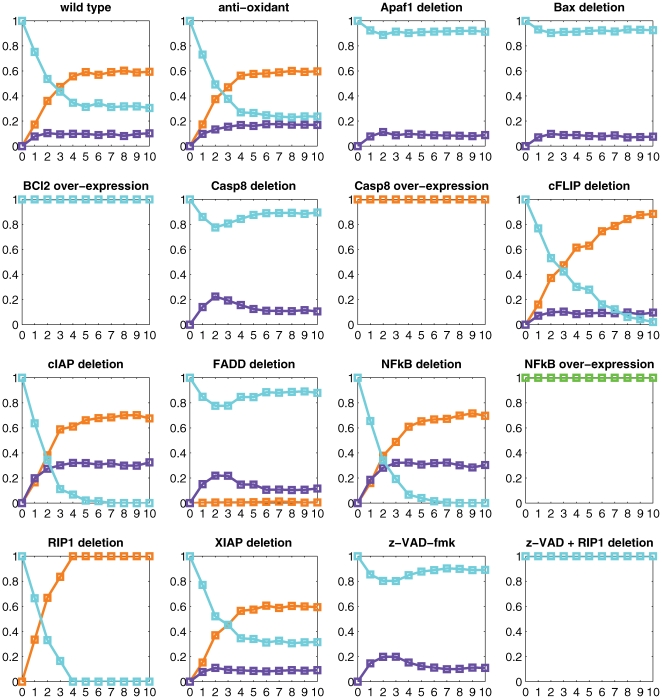
Ligand removal experiments. The x-axis represents the (discrete) duration of the TNF pulse *t_d_* (see text). At each discrete time point along the x-axis, the TNF signal is turned off. The different curves represent the average probabilities to reach the different attractors after the pulse (the number of trajectories *N* = 2000). Curves are coloured in blue for naïve state, green for NFκB survival, orange for apoptosis, and purple for necrosis.

In the reachability analysis presented above, the value of TNF and FASL are kept constant and therefore always ON (or always OFF) along all trajectories. These qualitative simulations are useful to characterize the *asymptotic* behaviour of the system when the death receptor is engaged for a sufficiently long time. The principle of ‘ligand removal’ experiments consists in characterizing the decision process when it is subject to a temporary pulse of TNF. Here, time is intrinsically discrete, meaning that the duration of TNF pulse denoted *t_d_* is represented by an integer number. In order to simulate each experiment, *N* trajectories were generated, starting from the “physiological” condition with TNF = 1. At time *t_d_*, the value of TNF is forced to zero. The probabilities to reach the different phenotypes are then calculated as explained in the Methods section. The average probabilities, over the *N* computed trajectories, are represented in Figure 6, for the wild type and the 15 mutants.

The purpose of this study is to investigate the dynamics of all the mutants and how they reach the various possible phenotypes for different lengths of TNF pulses. It provides a measurable way to assess the appearance or disappearance of certain phenotypes upon TNF induction. The curves of Figure 6 allow to link explicitly the graphs of Figure 4 when TNF is ON (right panels) and OFF (left panels) with the subjacent dynamics.

Let us compare the wild type case and the deletion of cFLIP as an example of how to read these graphs. For early events, the two cases behave similarly as expected (up to event 3). As TNF pulse is prolonged, the apoptotic phenotype becomes more and more pronounced and strongly favoured over the survival one in the cFLIP mutant as opposed to the wild type conditions. This leads to the complete disappearance of survival in the mutant. This observation reinforces the role of cFLIP in the control of the apoptotic pathway.

With the ‘ligand removal’ experiment, we can evaluate the number of steps, in the reduced model, that are needed for the cell to decide on its fate after TNF exposure. For almost all mutants and wild type case, the choice is made around step 4. This means that, after this point, even if TNF is removed, the cell has already committed to a specific fate.

One surprise arises from the non-monotonic behaviour of mutants for which apoptosis is suppressed (APAF1, BAX, caspase-8 and FADD deletions and z-VAD-fmk treatment), tentatively indicating a competition between components of the survival and necrotic pathways. Indeed several inhibitory cross-talks could explain this behaviour. These mutants also indicate the existence of an optimal TNF induction for which the maximum rate of necrosis is achieved (around step 2 in the corresponding mutants of Figure 6).

### A compact conceptual model

To complete our study of cell fate decision, we reasoned on the simplest model of cell fate that can be deduced from the master model described above.

The purpose here was to further simplify the network to obtain a formal representation of the logical core of the network. We have selected three components to represent the three cellular fates: NFκB for survival, MPT for necrosis and CASP3 for apoptosis. Based on reduction techniques and on the identification of all possible directed paths between these three components [50], a three-node diagram was deduced from the master model.

In this compact model, each original path (including regulatory circuit) is represented by an arc whose sign denotes the influence of the source node on its target. All original paths and the corresponding arcs are recorded in Table 3. In some ambiguous cases (e.g. influence of MPT on CASP3 or of NFκB on MPT), the decision on the sign of the influence is based on the Boolean rules and not on the paths only. Indeed, two negative and one positive paths link NFκB to MPT. Therefore, the sign of the arc depends not only on the states of BCL2 and of ROS, both feeding onto MPT, but also on the rule controlling MPT value. Since the absence of BCL2 *and* the presence of ROS (Boolean ‘AND’ gate) participate in the activation of MPT, if BCL2 is active, then MPT is set to 0, even when ROS is ON. By extension, if NFκB is ON, then MPT is 0, justifying the choice for a negative influence. In the case of mutations eliminating all the negative influences, however, a positive arrow must be considered.

**Table 3:** List of paths corresponding to single arcs in the conceptual model of Figure 7.

Arc type	Arc	Paths on the regulatory graph	Sign of regulation
*Feedback circuits*	**MPT⇒MPT**	1) MPT⇒ROS⇒MPT	(+)
	**NFκB⇒NFκB**	2) NFκB⇒cIAP⇒RIP1ub⇒IKK⇒NFκB	(+)
		3) NFκB⇒cFLIP⊣CASP8⊣RIP1⇒RIP1ub⇒IKK⇒NFκB	(+)
	**CASP3⇒CASP3**	4) CASP3⇒CASP8⇒BAX⇒MOMP⇒SMAC⊣XIAP⊣CASP3	(+)
		5) CASP3⇒CASP8⇒BAX⇒MOMP⇒Cyt_c⇒apoptosome⇒CASP3	(+)
*Other regulatory paths*	**CASP3⊣NFκB**	6) CASP3⇒CASP8⊣RIP1⇒RIP1ub⇒IKK⇒NFκB	(−)
		7) CASP3⇒CASP8⇒BAX⇒MOMP⇒SMAC⊣cIAP⇒RIP1ub⇒IKK⇒NFκB	(−)
		8) CASP3⊣NFκB	(−)
	**NFκB⊣CASP3**	9) NFκB⇒cFLIP⊣CASP8⇒BAX⇒MOMP⇒Cyt_c⇒apoptosome⇒CASP3	(−)
		10) NFκB⇒XIAP⊣CASP3	(−)
		11) NFκB⇒XIAP⊣Apoptosome⇒CASP3	(−)
		12) NFκB⇒BCL2⊣BAX⇒MOMP⇒Cyt_c⇒apoptosome⇒CASP3	(−)
	**MPT⊣NFκB**	13) MPT⇒MOMP⇒SMAC⊣cIAP⇒RIP1ub⇒IKK⇒NFκB	(−)
	**NFκB⊣MPT**	14) NFκB⊣ROS⇒MPT	(−)
		15) NFκB⇒BCL2⊣MPT	(−)
		16) NFκB⇒cFLIP⊣CASP8⊣RIP1⇒RIP1K⇒ROS⇒MPT	(+)
	**CASP3⊣MPT**	17) CASP3⇒CASP8⊣RIP1⇒RIP1K⇒ROS⇒MPT	(−)
	**MPT⊣CASP3**	18) MPT⇒MOMP⇒Cyt_c⇒apoptosome⇒CASP3	(+)
		19) MPT⇒MOMP⇒SMAC⊣XIAP⊣CASP3	(+)
		20) MPT⇒MOMP⇒SMAC⊣XIAP⊣apoptosome⇒CASP3	(+)
		21) MPT⊣ATP⇒apoptosome⇒CASP3	(−)

**Figure 7:**
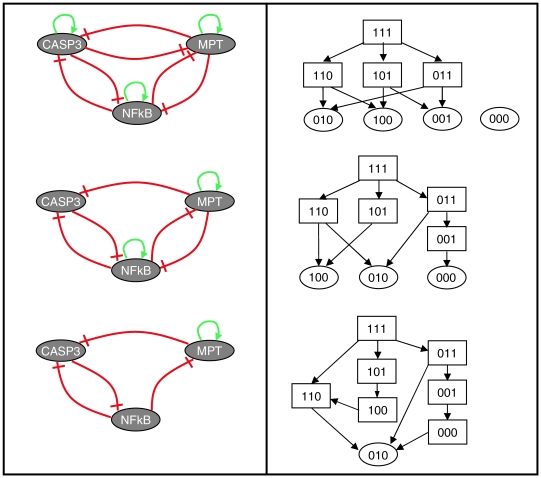
Simplified view of the cell fate model structure. *Left panels*: compact regulatory graph deduced from the master model (top), along with two variants (middle and low). *Right panels*: state transition graphs corresponding to each regulatory graph, using generic logical rules (cf. text). Stable states are represented by ellipses (at the bottom of each state transition graph). Each stable state corresponds to one cell fate: 000 for the ‘naïve’ state, 010 for survival, 001 for apoptosis, and 100 for necrosis. *Top*: wild type structure. *Middle*: *CASP8* deletion mutant. *Bottom*: *CASP8* deletion mutant in the absence of cIAP.

The resulting molecular network is symmetrical: each node is self-activating and is negatively regulated by the other nodes (Figure 7, upper left panel). This is a conceptual picture representing the general architecture of the master model that can help address specific questions. Even for this relatively simple regulatory graph, there is a finite but quite high number of possible logical rules. For now, we use a simple generic rule involving the AND and NOT operators. For example, the logical rule for CASP3 is: NOT MPT AND NOT NFkB AND CASP3. This compact model has four stable states, each corresponding to one cell fate, along with the ‘naïve’ state (Figure 7, upper right panel). This is coherent with what was observed from the analysis of the complete model.

To validate our compact model, we verified that the simulations of known mutations correspond to the published observations. Here, when a hidden component is deleted, all the paths traversing this component in the original graph are broken. If all the paths corresponding to an arc of the compact model happen to be broken, then it is removed. In the case of auto-regulation, not only the link is broken but the node is also set to zero to avoid the node to become active in the absence of death receptor activation.

Let us consider the *CASP8* deletion mutant to illustrate this approach (Figure 7, middle panels). For this mutant, several arrows in the compact model have to be deleted. For example, the arcs CASP3⇒CASP3 (paths 4+5 in Table 3) and CASP3⊣MPT (path 17) clearly depend on the activation of CASP8. Note, however, that CASP8 intervenes in other paths, which do not fully rely on its sole activity. In the case of the arc NFκB⇒NFκB, CASP8 depletion interrupts path 3, while path 2 can still enable the NFκB auto-regulation. Consistent with the results from the previous section, CASP8 depletion leads to the loss of the apoptotic fate while the ‘naïve’ stable state cannot be attained.

At this point, one could wonder how apoptosis could be re-established in a *CASP8* mutant. The analysis of the broken paths suggests some experiments to bypass CASP8 and undergo CASP3 activation. On the basis of path 4, BAX, MOMP, SMAC and XIAP are identified as potential targets, while path 5 points to cytochrome c and apoptosome. One way to experimentally assess this possibility would be to inject exogenous cytochrome c as it was done in ‘wild type’ conditions [51], or yet provoke its release from the mitochondria by forcing the opening of the pores. This is possible only in the absence (or with low activity) of NFκB and in the presence of ATP. Again, since no quantitative information can be deduced from the path analysis proposed in this study, no prediction can be made on the concentrations of proteins needed to achieve a specific answer.

In a previous section, we postulated that an inhibition of the survival pathway by the necrotic pathway is necessary to reproduce some mutant phenotype. We suggested that cIAP could play this role. Let us now test this hypothesis with our conceptual model. We build the corresponding 3-node model without cIAP. In the current version, cIAP plays two important roles, first as a mandatory intermediate in the inhibitory effect of MPT (associated with necrosis) onto NFκB (survival) (path 13), next as an obligate intermediate in the self-activation of NFκB (path 2). The simulation (Figure 7, lower right panel) shows that in the absence of cIAP, it is impossible to obtain the necrosis cell fate in the *CASP8* (and *FADD*) mutant(s), in agreement with our previous conclusions and in support of our suggestion. A complete list of all possible gene knockouts is provided in the Table S2.

This conceptual model analysis underlines the importance to simplify in order to better understand the general structure of the network and reason on it. Indeed, the simple 3-node network enables us to grasp global functional aspects and propose specific qualitative predictions.

## Discussion

Mathematical models provide a way to test biological hypotheses *in silico*. They recapitulate consistent heterogeneous published results and assemble disseminated information into a coherent picture using a coherent mathematical formalism (discrete, continuous, stochastic, hybrid, etc.), depending on the questions and the available data. Then, modelling consists of constantly challenging the obtained model with available published data or experimental results (mutants or drug treatments). After several refinement rounds, a model becomes particularly useful when it can provide counter-intuitive insights or suggest novel promising experiments.

Here, we have conceived a mathematical model of cell fate decision, based on a logical formalisation of well-characterised molecular interactions. Former mathematical models only considered two cellular fates, apoptosis and cell survival. In contrast, we include a non-apoptotic modality of cell death, mainly necrosis, involving RIP1, ROS and mitochondria functions.

Both the master and the reduced models were constructed on the basis of an extensive analysis of the literature. The master model (Figure 1A) summarises our current understanding of the mechanisms regulating cell fate decision and identifies the major switches in this decision. However, some important interactions, components (caspase-2, calpains, AIF, etc.) or pathways (JNK, Akt, etc.) have not yet been considered. This model was built to be as generic as possible. Most of the mutants considered were analysed in Jurkat cells, T-cells, or L929 murine fibrosarcoma cells, thus in very different cellular contexts (e.g. in response to TNF, Jurkat cells are resistant to cell death, whereas L929 cell lines undergo necrosis). We are trying to account for all those phenotypes in a unique model. The next step will be to provide a model variant for each cell type in order to better match cell-specific behaviours.

The reduced models can be used to simulate observed experiments and to reflect on the general mechanisms involved in apoptosis, survival or necrosis. This led us to identify the principal actors involved in the decision process. The presence of RIP1 or FADD, for example, proved to be decisive in our simulation. However, the role of cFLIP appears less obvious than previously suggested [7].

We can easily perturb the structure of the system *in silico* and assess the dynamical effects of such perturbations (e.g. novel knockouts). Our model can also be used to decide between antagonist results found in different publications. For instance, the inhibitory role of cIAP1/2 on the apoptotic pathway was initially attributed to a direct inhibition of caspases. However, detailed biochemical studies challenged this view [52],[53]. We have tested this hypothesis by adding an inhibitory arc from cIAP onto CASP8, but simulations do not support a functional inhibitory role of cIAP1/2, since survival is favoured over apoptosis in many mutants, thus making apoptosis a very improbable phenotype (Figure S1). Similarly, we tested the role of the feedback circuit involving CASP8 and CASP3. We found that the activation of CASP8 by CASP3 is not functional when TNF and FASL are constantly ON. However, when TNF or FAS signal is not sustained, CASP3⇒CASP8 activation becomes necessary to insure the persistence of the apoptotic phenotype. When TNF is sustained, this feedback is no longer needed (see Figures S2 and S3 for details).

The in-depth analysis of model properties led us to propose several predictions or novel insights. Some concern the structure of the network, as several interactions appear to be necessary to achieve specific phenotypes. For example, our simulations of *FADD* and *CASP8* deletion mutants underline the need for a mechanism from the necrotic pathway that would inhibit the survival one. Here, we consider a mechanism involving MPT, SMAC and cIAP. Other simulations point to different roles of proteins: RIP1 activity is transient in necrosis whereas it is sustained in survival. Similarly, our model analysis shows the role played by the duration of the TNF pulses in the cell fate decision and enlights when this decision is made. Finally, some hints about possible scenarios for forcing or restoring a phenotype in mutants are provided.

Deregulations of the signalling pathways studied here can lead to drastic and serious consequences. Hanahan and Weinberg proposed that escape of apoptosis, together with other alterations of cellular physiology, represents a necessary event in cancer promotion and progression [54]. As a result, somatic mutations leading to impaired apoptosis are expected to be associated with cancer. In the cell fate model presented here, most nodes can be classified as pro-apoptotic or anti-apoptotic according to the results of “mutant” model simulations, which are correlated with experimental results found in the literature. Genes classified as pro-apoptotic in our model include caspases-8 and -3, APAF1 as part of the apoptosome complex, cytochrome c (Cyt_c), BAX, and SMAC. Anti-apoptotic genes encompass BCL2, cIAP1/2, XIAP, cFLIP, and different genes involved in the NFκB pathway, including NFKB1, RELA, IKBKG and IKBKB (not explicit in the model). Genetic alterations leading to loss of activity of pro-apoptotic genes or to increased activity of anti-apoptotic genes have been associated with various cancers. Thus, we can cross-list the alterations of these genes deduced from the model with what is reported in the literature and verify their role and implications in cancer.

For instance, concerning pro-apoptotic genes, frameshift mutations in the ORF of the BAX gene are reported in >50% of colorectal tumours of the micro-satellite mutator phenotype [55]. Expression of CASP8 is reduced in ∼24% of tumours from patients with Ewing's sarcoma [56]. Caspase-8 was suggested in several studies to function as a tumour suppressor in neuroblastomas [57] and in lung cancer [58].

On the other hand, constitutive activation of anti-apoptotic genes is often observed in cancer cells. The most striking example is the over-expression of the BCL2 oncogene in almost all follicular lymphomas, which can result from a t(14;18) translocation that positions BCL2 in close proximity to enhancer elements of the immunoglobulin heavy-chain locus [59]. As for the survival pathway, elevated NFκB activity, resulting from different genetic alterations or expression of the v-rel viral NFκB isoform, is detected in multiple cancers, including lymphomas and breast cancers [60]. An amplification of the genomic region 11q22 that spans over the cIAP1 and cIAP2 genes is associated with lung cancers [61], cervical cancer resistance to radiotherapy [62], and oesophageal squamous cell carcinomas [63].

A better understanding of the pro- or anti-apoptotic roles of these genes involved in various cancers and their interactions with other pathways would set a ground for re-establishing a lost death phenotype and identifying druggable targets. The cell fate model proposed here is a first step in this direction.

In the future, we will consider additional signalling cascades and their cross-talks, following the path open by other groups [64]. In parallel, we are contemplating the inclusion of other modalities of cell death such as autophagy [65], which inhibits apoptosis through BCL2 and is itself inhibited by apoptosis through Beclin1. The functioning of the intrinsic apoptotic pathway and the internal cellular mechanisms capable of triggering it could be investigated in more details, taking advantage of recent molecular analyses [66],[67]. Finally, when systematic quantitative data regarding the decision between multiple cell fates will become available, our qualitative model could be used to design more quantitative models adapted to specific cellular systems in order to predict the probability for a given cell to enter into a particular cell fate depending on stimuli.

## Methods

### Boolean formalism, synchronous vs. asynchronous strategy

The computation of trajectories in the state space consists in the calculation of sequences of states where each member of the sequence is a *logical successor* of the previous one. As we choose to use Boolean variables to encode the 25-dimensional master model, the state space is the set *S* = {0,1}^25^. Although finite, the size of this set is huge (more than 33 millions states). Furthermore, in the discrete framework, the mathematical definition of the trajectories assumes an updating rule for the variables. Two main strategies are usually considered to analyse discrete models of biological networks. The first one consists in updating *all* variables simultaneously, at each time step. This synchronous strategy [68] has the advantage to generate simple determinist dynamics, each state having one and only one successor. Drawing a directed arrow from each of the 2^25^ states to its successor, one constructs the synchronous transition graph, comprising all synchronous trajectories of the system. The determinism of the synchronous transition graph is a very strong property that poorly portrays the complexity of the biochemical processes that are modelled (some processes are likely to occur faster than others). The second strategy, which is used in this paper, consists in considering that only one component is updated at each time, implying that a state may have several successors [69].

More precisely, to compute the set of asynchronous successors of a state *x* = (*x*_1_,…,*x*_n_)∈{0,1}^n^, one has to follow the three steps: (1) compute the state *F*(*x*) = (*f*_1_(*x*),…, *f*_n_(*x*)), where *f_i_* is the Boolean rule of the *i* ^th^ variable (*F(x)* is thus the synchronous successor of *x*); (2) select the indices *i* such that *x*_i_≠*f*_i_(*x*) (those are the indices of the variables that are liable to change when the system is in state *x*); and (3) for all such indices *i*, the state (*x*_1_,…,*f*_i_(*x*),…,*x*_n_) is an asynchronous successor of *x*.

According to this definition, in the asynchronous approach, no *a priori* hypothesis is made on the order of the events: all possible orders are considered, which is much more satisfying from a modelling point of view, as it is very difficult to know the relative speeds of the different processes involved in the master model. Note that the stable states of the model are independent on the choice of the strategy (synchronous or asynchronous). Therefore, the first analysis (based on the clustering of stable states) is valid regardless the updating strategy.

Drawing an arrow from each state to its asynchronous successors leads to the construction of an asynchronous transition graph, which comprises all possible asynchronous trajectories of the system. To each arrow starting from the same state is associated an equal probability (see [70] for details). This is a strong assumption, which is the main reason why the exact values of computed probabilities (of the different phenotypes) should not be compared to experimental data in a quantitative manner. Nevertheless, the same assumption has been made for all model variants (mutants and drug treatments), thereby allowing comparative studies. A systematic method to assess the impact of the probability distribution is a key point towards a finer quantitative analysis (work in progress).

As pointed earlier, the size of the transition graph is exponential with respect to the number of variables, which constitutes a first obstacle to the dynamical analysis. A second difficulty resides in the fact that the asynchronous graph is not deterministic, as each vertex may have more than one successor, which, given the size of the graph, makes the application of classical graph algorithms computationally heavy.

### Model reduction

We have used a model reduction technique specifically adapted to discrete systems, which mainly consists in iteratively “hiding” some variables, while keeping track of underlying regulatory processes [47]. The main dynamical properties of the master model, including stable states and other attractors are conserved in the reduced model. Thanks to the computation of the reduced asynchronous transition graph, relevant qualitative dynamical properties of the model can be compared to experimental results for wild type and in different mutant cases.

To reduce the number of species in the master model, each logical rule is considered. For each removed component, the information contained in its rule is included in the rules of its targets such that no effective regulation is lost.

Many intermediate components could easily be replaced by a proper rewriting of the logical rules associated with their target nodes. For example, IKK has only one input (RIP1ub) and one output (NFκB). Since its role in our model merely consists in transmitting the signal from RIP1ub to NFκB, it can be easily replaced by a straightforward change in the logical rule associated with NFκB (implementing a direct activation from RIP1ub instead of IKK). We also relied on the results of the clustering of stable states and their associations with biologically plausible phenotypes to select the key components to keep in the reduced model: NFκB is the principal survival actor, while caspases-3 and -8, together with the mitochondrial membrane permeability variables (MOMP and MPT), determine apoptotic and non-apoptotic cell deaths.

Let us consider the example of the removal of BAX and BCL2 (Figure 1 A and B). The regulators (or inputs) of these variables are NFκB for BCL2 and CASP8 for BAX while their regulating targets (or outputs) are MPT for BCL2 and MOMP for BAX. BCL2 is directly activated by NFκB, and has two targets: MPT and BAX. Therefore, BCL2 removal is performed by replacing BCL2 by NFκB into the rules of the two targets, leading to the two new logical rules: MPT′ = ROS AND NOT NFkB and BAX′ = C8 AND NOT NFkB. Applying the same process to remove BAX, one obtains the following new rule for MOMP: MOMP′ = MPT OR (C8 AND NOT NFkB).

The variables MOMP and MPT have now as inputs the variables NFκB and CASP8. One can see that, in spite of the disappearance of variables BAX and BCL2, their regulating roles are still indirectly coded in the reduced system, ensuring that no “logical interaction” of the master model (i.e. activation or inhibition) is actually lost during the reduction process. Table S3 lists the variables of the master model that are removed to obtain the reduced model.

Some hypotheses were made when reducing the model. First, FADD is considered to be constantly ON in wild type simulations. Second, since the two complexes TNFR and DISC-TNF have been removed together with the input FADD, the two deaths ligands TNF and L have the exact same action in the reduced model. Indeed, we consider that, in response to FAS death receptor engagement as well as that of TNF; the activations of both the survival and necrotic pathways RIP1-dependent. In this case, one could then merge these variables and consider only one input that could be called “external death receptor”. However, we choose to keep the two variables TNF and FASL, in the *FADD* deletion mutant, the phenotype differs for TNF and FAS signal: actually, only for that mutant is the symmetry of TNF and FAS broken.

## Supporting Information

Figure S1 Simulations of the reduced cell fate model incremented by the interaction cIAP⊣CASP8 with TNF = 1, FasL = 0.
(0.10 MB PDF)

Figure S2 Simulations of the model after deletion of the interaction CASP3⇒CASP8 from the model with TNF = 1, FasL = 0.
(0.11 MB PDF)

Figure S3 Ligand removal simulation for the model with the deletion of the interaction CASP3⇒CASP8.
(0.08 MB PDF)

Figure S4 Comparative simulations between master model and reduced model.
(0.13 MB PDF)

Table S1 Contribution of the different variables into the first two principal components. The colour marks relatively large contributions, positive in red and negative in green.
(0.01 MB PDF)

Table S2 List of mutants simulated with the conceptual 3-node model.
(0.03 MB PDF)

Table S3 List of variables hidden from the master model to generate the reduced model.
(0.07 MB PDF)

Text S1 Supplementary Text includes 1) GINsim Report of the Annotated Model and 2) Supplementary references
(0.16 MB PDF)
