# Supplementary material for: Mathematical Modelling of Cell-Fate Decision in Response to Death Receptor Engagement
Source: PLoS Comput Biol. 2010 Mar 5;6(3):e1000702. doi: 10.1371/journal.pcbi.1000702 (PMC2832675; doi:10.1371/journal.pcbi.1000702)
Supplement: Figure S1 — Simulations of the reduced cell fate model incremented by the interaction cIAP⊣CASP8 with TNF = 1, FasL = 0. (0.10 MB PDF) [file pcbi.1000702.s001.pdf]

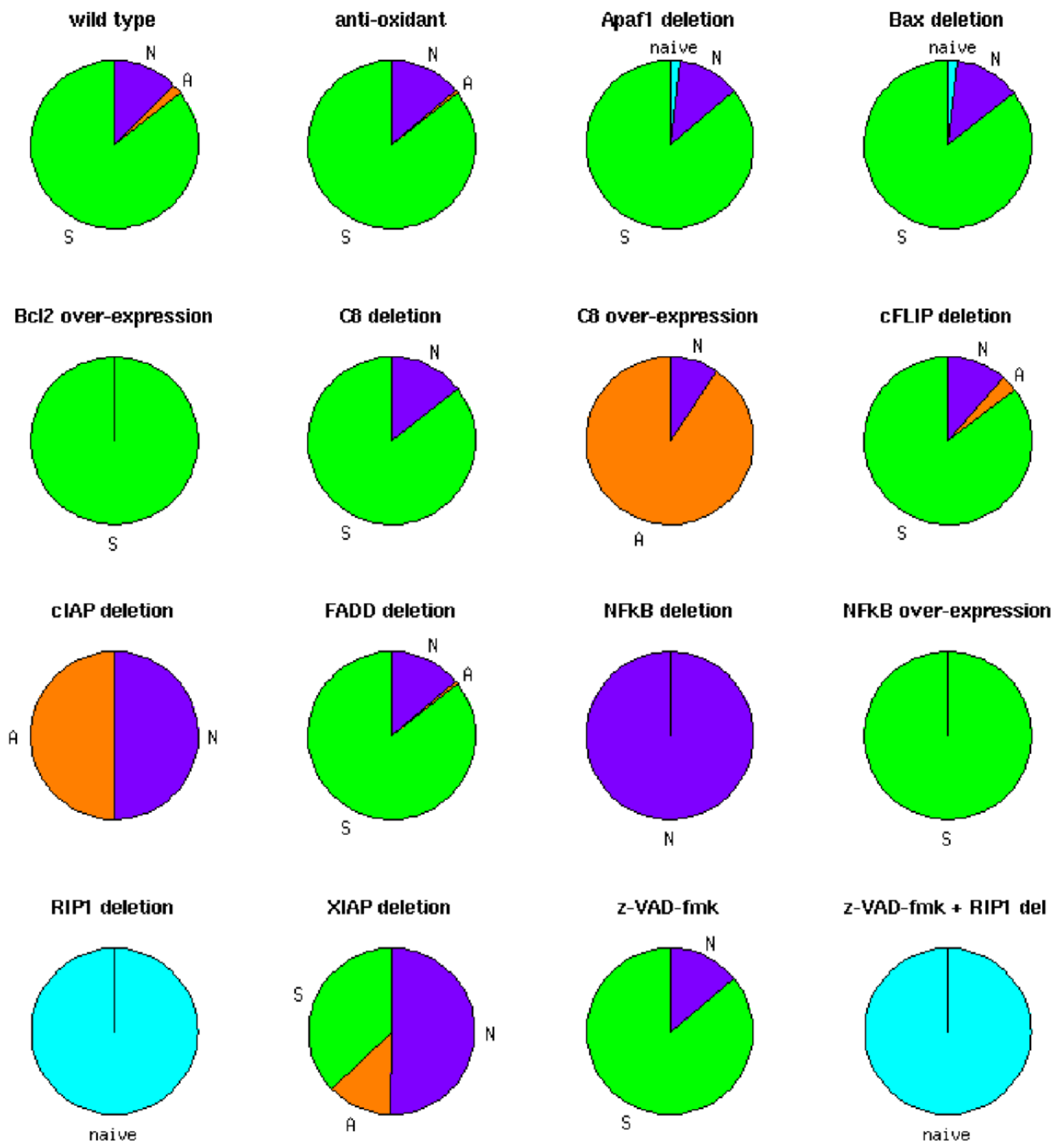

Supp. Figure S1. Simulations of the reduced cell fate model incremented by the interaction cIAP -| CASP8 with TNF=1, FasL=0.
