# Supplementary material for: Mathematical Modelling of Cell-Fate Decision in Response to Death Receptor Engagement
Source: PLoS Comput Biol. 2010 Mar 5;6(3):e1000702. doi: 10.1371/journal.pcbi.1000702 (PMC2832675; doi:10.1371/journal.pcbi.1000702)
Supplement: Figure S2 — Simulations of the model after deletion of the interaction CASP3⇒CASP8 from the model with TNF = 1, FasL = 0. (0.11 MB PDF) [file pcbi.1000702.s002.pdf]

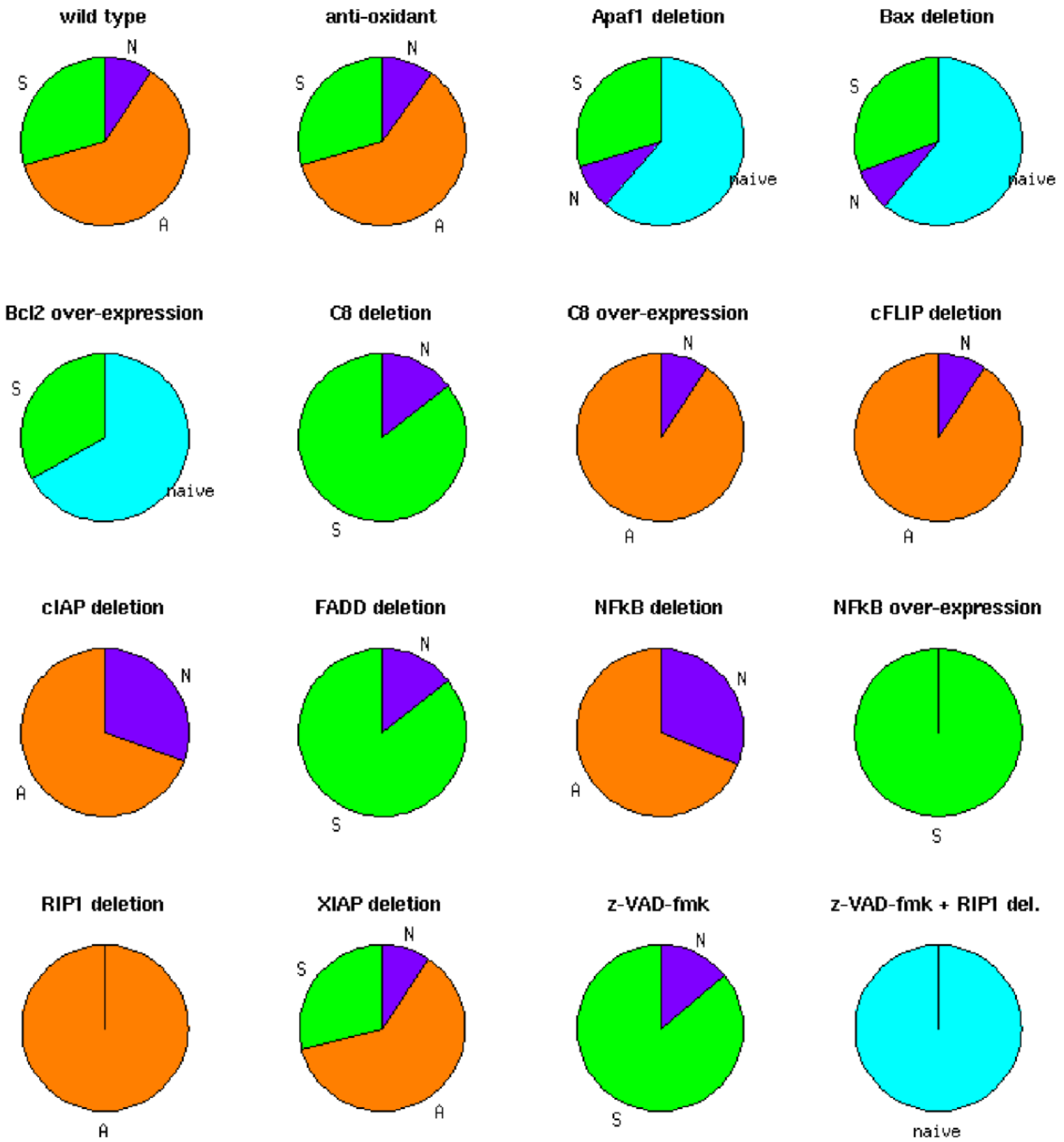

Supp. Figure S2. Simulations of the model after deletion of the interaction CASP3 => CASP8 from the model with TNF=1, FasL=0.
