# Supplementary material for: Mathematical Modelling of Cell-Fate Decision in Response to Death Receptor Engagement
Source: PLoS Comput Biol. 2010 Mar 5;6(3):e1000702. doi: 10.1371/journal.pcbi.1000702 (PMC2832675; doi:10.1371/journal.pcbi.1000702)
Supplement: Figure S3 — Ligand removal simulation for the model with the deletion of the interaction CASP3⇒CASP8. (0.08 MB PDF) [file pcbi.1000702.s003.pdf]

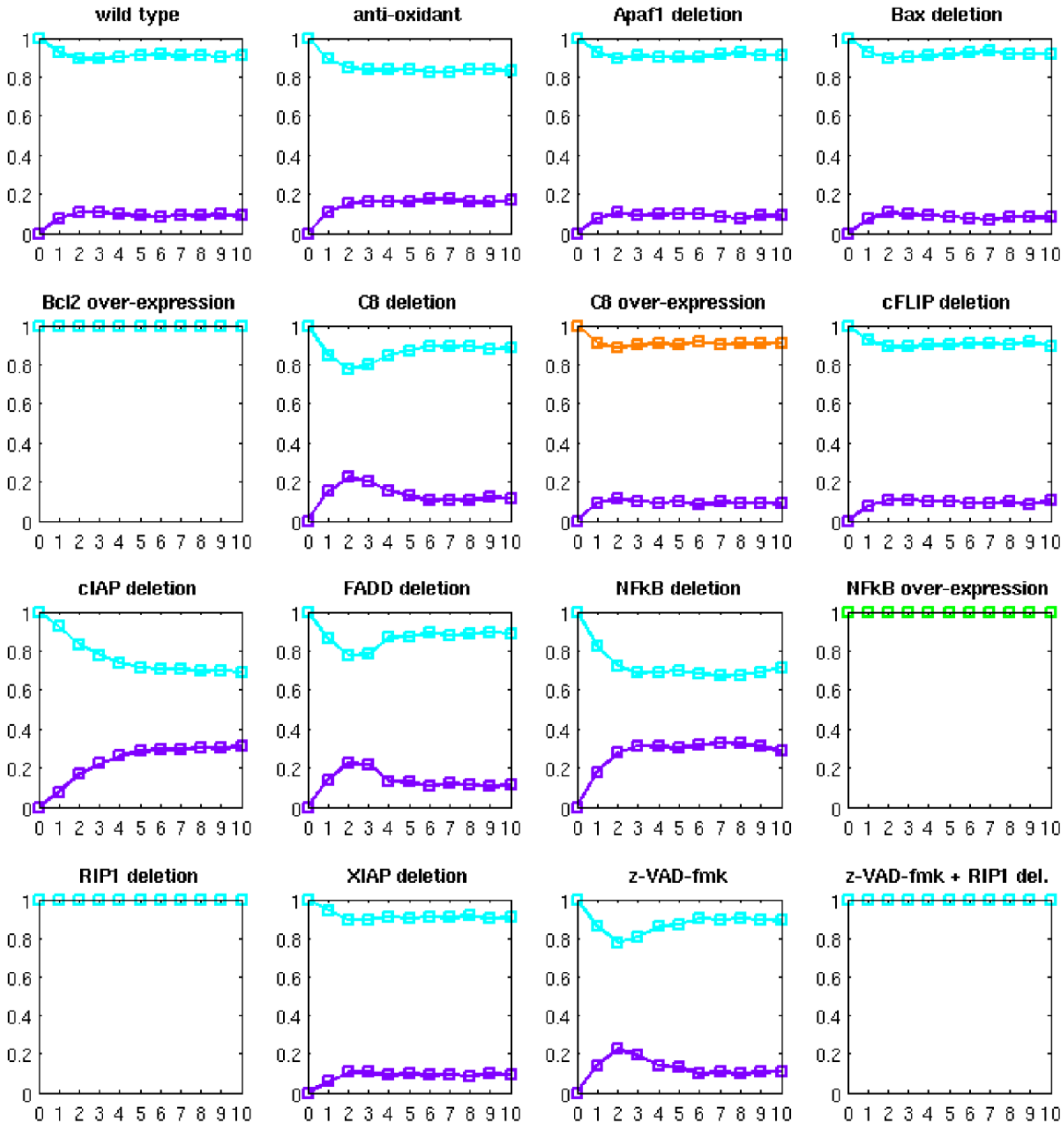

Supp. Figure S3. Ligand removal simulation for the model with the deletion of the interaction CASP3 => CASP8.
