# Supplementary material for: Mathematical Modelling of Cell-Fate Decision in Response to Death Receptor Engagement
Source: PLoS Comput Biol. 2010 Mar 5;6(3):e1000702. doi: 10.1371/journal.pcbi.1000702 (PMC2832675; doi:10.1371/journal.pcbi.1000702)
Supplement: Figure S4 — Comparative simulations between master model and reduced model. (0.13 MB PDF) [file pcbi.1000702.s004.pdf]

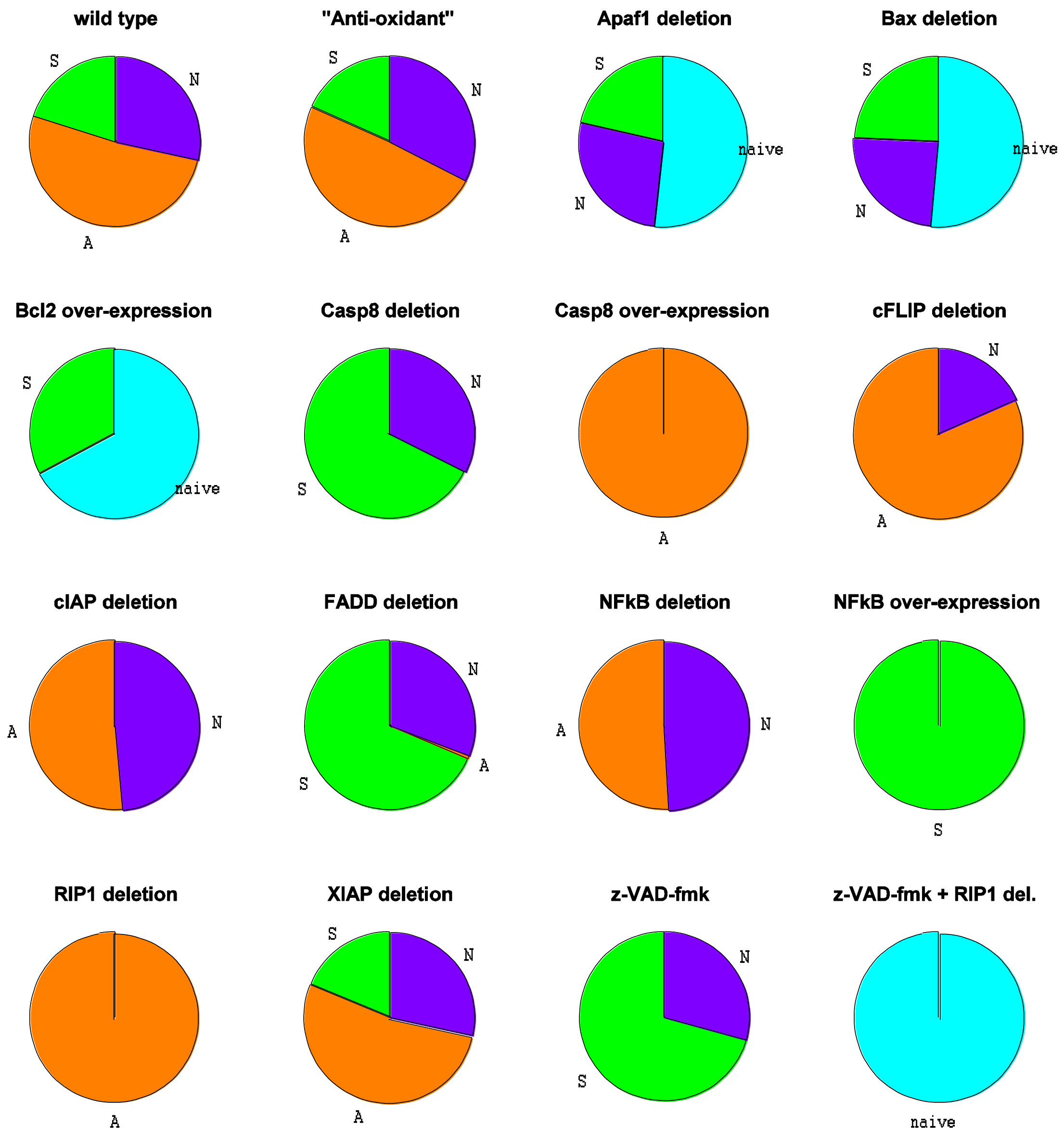

Fig. S4. Comparative simulations between master model and reduced model.

To assess the effect of the model reduction onto the relative probability to reach the different attractors, we developed a sampling strategy to be applied to the master model and to the corresponding "mutants", starting from the physiological initial condition and perturbed by TNFR engagement. For each model, 10000 trajectories were randomly generated and the corresponding attractors classified as apoptosis (when they displayed CASP3=1), necrosis (ATP=0), survival with NFκB (NFκB=1), or naïve survival (none of the previous conditions fulfilled). Indeed, neither attractor displaying 2 out of these 3 conditions, nor any cyclic attractor, were reached, rendering this classification straightforward and consistent with the reduced model.

This figure shows the probabilities to reach the different classes of attractors calculated using this sampling procedure. If the exact numerical values differ for almost all "mutants" between the master model (this figure) and the reduced model (Figure 3 of the main text), the qualitative behaviours of these models are equivalent. First, for every "mutant" the reachable phenotypes are exactly the same, as even the very few trajectories that lead to an apoptotic phenotype in the *FADD* mutant are observed in both versions. Then, the repartition of phenotypes between any two mutants evolves in the same direction, that is to say, if the probability for a phenotype increases when compared to the wild type in the reduced model, it increases as well in the master model. Finally, these considerations illustrate that the qualitative properties of the model are conserved upon model reduction.
