# Supplementary material for: Mathematical Modelling of Cell-Fate Decision in Response to Death Receptor Engagement
Source: PLoS Comput Biol. 2010 Mar 5;6(3):e1000702. doi: 10.1371/journal.pcbi.1000702 (PMC2832675; doi:10.1371/journal.pcbi.1000702)
Supplement: Table S1 — Contribution of the different variables into the first two principal components. The colour marks relatively large contributions, positive in red and negative in green. (0.01 MB PDF) [file pcbi.1000702.s005.pdf]

	PC1	PC2
Cyt_c	-0.25	-0.04
MOMP	-0.25	-0.04
SMAC	-0.25	-0.04
Bax	-0.20	0.26
CASP8	-0.20	0.26
Apoptosome	-0.16	0.38
CASP3	-0.16	0.38
MPT	-0.11	-0.42
ROS	-0.11	-0.42
C2_TNF	0.02	0.06
DISC_FAS	0.02	0.06
ATP	0.11	0.42
RIP1	0.24	-0.10
RIP1K	0.24	-0.10
cIAP	0.26	0.04
Bcl2	0.28	0.05
cFLIP	0.28	0.05
IKK	0.28	0.05
NFkB	0.28	0.05
RIP1ub	0.28	0.05
XIAP	0.28	0.05

Supp. Table S1. Contribution of the different variables into the first two principal components. The colour marks relatively large contributions, positive in red and negative in green.
