# Supplementary material for: Mathematical Modelling of Cell-Fate Decision in Response to Death Receptor Engagement
Source: PLoS Comput Biol. 2010 Mar 5;6(3):e1000702. doi: 10.1371/journal.pcbi.1000702 (PMC2832675; doi:10.1371/journal.pcbi.1000702)
Supplement: Table S2 — List of mutants simulated with the conceptual 3-node model. (0.03 MB PDF) [file pcbi.1000702.s006.pdf]

Mutants	changes in the structure - deleted paths	phenotypes	comments
anti-oxidants	no changes	000 (naive)<br>001 (necrosis)<br>010 (survival)<br>100 (apoptosis)	similar to WT
APAF1 del	CASP3 => CASP3	000 (naive)<br>001 (necrosis)<br>010 (survival)	loss of apoptosis, equivalent to a deletion of CASP3
Bax del	CASP3 => CASP3 (paths 4, 5)	000 (naive)<br>001 (necrosis)<br>010 (survival)	loss of apoptosis
Bcl2 op	MPT =0 & CASP3 => CASP3 (paths 4, 5)	000 (naive)<br>010 (survival)	loss of apoptosis and necrosis
Casp8 del	CASP3 => CASP3 (paths 4, 5) & CASP3 -| MPT (path 17)	000 (naive)<br>001 (necrosis)<br>010 (survival)	loss of apoptosis
Casp8 op	NFkB => NFkB (paths 2, 3) & CASP3 -| MPT (path 17) & MPT -| NFkB (path 13)	000 (naive)<br>001 (necrosis)<br>100 (apoptosis)	loss of survival, similar to cFLIP del and RIP1 del
cFLIP del	NFkB => NFkB (paths 2, 3) & CASP3 -| MPT (path 17) & MPT -| NFkB (path 13)	000 (naive)<br>001 (necrosis)<br>100 (apoptosis)	loss of survival, similar to Casp8 op and RIP1 del
cIAP del	NFkB => NFkB (paths 2, 3) & MPT -| NFkB (path 13)	000 (naive)<br>001 (necrosis)<br>100 (apoptosis)	loss of survival
FADD del	CASP3 => CASP3 (paths 4, 5) & CASP3 -| MPT (path 17)	000 (naive)<br>001 (necrosis)<br>010 (survival)	loss of apoptosis
NFkB del	NFkB=0 (paths 2, 3)	000 (naive)<br>001 (necrosis)<br>100 (apoptosis)	loss of survival
NFkB op	CASP3 => CASP3 MPT => MPT	000 (naive)<br>010 (survival)	loss of cell death
RIP1 del	NFkB => NFkB (paths 2, 3) & CASP3 -| MPT (path 17) & MPT -| NFkB (path 13)	000 (naive)<br>001 (necrosis)<br>100 (apoptosis)	loss of survival, similar to Casp8 op and cFLIP del (In the full model, necrosis disappears as well)
XIAP del	no changes	000 (naive)<br>001 (necrosis)<br>010 (survival)<br>100 (apoptosis)	similar to WT
z-VAD-fmk	CASP3 => CASP3	000 (naive)<br>001 (necrosis)<br>010 (survival)	loss of apoptosis

Supp. Table S2. List of mutants simulated with the conceptual 3-node model.
