# Supplementary material for: Mathematical Modelling of Cell-Fate Decision in Response to Death Receptor Engagement
Source: PLoS Comput Biol. 2010 Mar 5;6(3):e1000702. doi: 10.1371/journal.pcbi.1000702 (PMC2832675; doi:10.1371/journal.pcbi.1000702)
Supplement: Table S3 — List of variables hidden from the master model to generate the reduced model. (0.07 MB PDF) [file pcbi.1000702.s007.pdf]

| **Removed variables** | **Justification** |
|---|---|
| FADD | In wild type, the input FADD is only relevant when it is equal to 1. The case where FADD=0 will be considered later as a “mutant case”. |
| TNFR, DISC-TNF | DISC-TNF (Complex II) is directly activated by TNFR, which is itself directly activated by TNF. The influence of these two variables is replaced by a direct activation by TNF (losing the necessity of forming Complex II to activate the apoptosis pathway via TNF-induced signal). |
| DISC-FAS | For similar reasons, DISC-FAS is removed and replaced by a direct activation by FasL. |
| RIP1K, RIP1ub, IKK | RIP1K, RIP1ub, and IKK influences are replaced by RIP1 itself. |
| cFLIP | cFLIP is replaced by a direct inhibition of NFkB on caspase-8. |
| BCL2, BAX | The actions of BCL2 and BAX are included in the rules of MOMP (with inputs from NFkB) and of MPT for BCL2 |
| XIAP, Cyt_c, apoptosome | The actions of these variables are included in the rule of caspase-3. |
| SMAC | SMAC rule is included in the rule of cIAP. |

Supp. Table S3. List of variables hidden from the master model to generate the reduced model.
