# Supplementary material for: Mathematical Modelling of Cell-Fate Decision in Response to Death Receptor Engagement
Source: PLoS Comput Biol. 2010 Mar 5;6(3):e1000702. doi: 10.1371/journal.pcbi.1000702 (PMC2832675; doi:10.1371/journal.pcbi.1000702)
Supplement: Text S1 — Supplementary Text includes 1) GINsim Report of the Annotated Model and 2) Supplementary references (0.16 MB PDF) [file pcbi.1000702.s008.pdf]

SUPPLEMENTARY MATERIAL

Mathematical Modelling of Cell-Fate Decision in Response to Death Receptor Engagement

Laurence Calzone[1,2,3], Laurent Tournier[1,2,3], Simon Fourquet[1,2,3], Denis Thieffry[4], Boris Zhivotovsky[5], Emmanuel Barillot[1,2,3,*], and Andrei Zinovyev[1,2,3,*]

[1] Institut Curie, Paris, France
[2] Ecole des Mines ParisTech, Paris, France
[3] INSERM U900, Paris, France
[4] TAGC – INSERM U928 & Université de la Méditerranée, 13288 Marseille Cedex 09
[5] Karolinska Institutet, SE-171 77 Stockholm, Sweden
[*] joint senior authorship

GINsim REPORT OF THE ANNOTATED MODEL 2
SUPPLEMENTARY REFERENCES 5

GINsim REPORT OF THE ANNOTATED MODEL

ID	Value	Logical function	Comment
FASL	1	FASL:1	
TNF	1	TNF:1	
TNFR	1	TNF:1	TNFR represents the engagement of TNF receptor by TNF.
DISC-TNF	1	FADD:1 & TNFR:1	DISC-TNF (TNF complex II) is a protein complex that acts as a signalling platform that assembles following TNFR1 engagement. It is composed of TRADD, TRAF2, FADD and caspase-8 but not TNFR1 [1].
DISC-FAS	1	FASL:1 & FADD:1	DISC-FAS is a protein complex that acts as a signalling platform. It is formed by recruitment of at least FADD and pro-CASP8 at the intracellular domain of FAS following engagement of FAS by FASL [2].
FADD	1	FADD:1	FADD represents the presence of protein FADD, that acts as an adaptor in death domain receptors complexes TNF-complex II (DISC-TNF) [1] and DISC-FAS [2].<br><br>FADD:0 represents conditions of genetic invalidation of FADD.
RIP1	1	(DISC-FAS:1 | TNFR:1) & !CASP8	RIP1 represents the recruitment of RIP1 protein to death receptor associated complexes TNFR1-complex I [1] and DISC-FAS [3,4].<br><br>Active CASP8 cleaves RIP1, leading to inhibition of NFkB mediated antiapoptotic signalling [5,6] and RIP1-mediated necrosis<br>[7].
RIP1ub	1	cIAP:1 & RIP1:1	RIP1ub represents the K63-linked ubiquitin conjugation of RIP1.<br><br>IAPs act as a RIP1 E3-ubiquitin ligases. [8,9]
RIP1K	1	RIP1:1	RIP1K represents to the kinase activity of RIP1<br><br>FAS triggers a caspase-8-independent cell death pathway using the kinase RIP as effector molecule. The kinase activity of RIP1 is indispensable [3].
IKK	1	RIP1ub:1	RIP1 K63-ubiquitinated within complex I recruits member of the IKK complex NEMO upstream of NF-kB activation [5].<br><br>RIP mediates the TNF-induced [10] and FAS-induced [4] NFkB activation.<br><br>The kinase activity of RIP1 is not required for TNF-induced IKK activation [11].
NFkB	0<br><br>1	CASP3:1<br><br>IKK:1 & !CASP3	In the so-called canonical pathway, NFkB is activated by release from its inhibitor IkB, which arises following IKK mediated phosphorylation of IkB [12].<br><br>Several components in the canonical NFkB activation pathway are substrates for CASP3-mediated proteolysis

			[13].
CASP8	0<br><br>1	cFLIP<br><br>(DISC-TNF | DISC-FAS | CASP3:1) & !cFLIP	cFLIP inhibits the activation of caspase-8 at the receptor level [14].<br><br>TNF complex II recruits CASP8 which leads to its activation [1].<br><br>CASP8 is activated by self-cleavage upon recruitment into the DISC-FAS complex [15,16].<br><br>In a positive feed-forward amplification loop, CASP3 proteolytically activates CASP8, with CASP6 possibly acting as an intermediate in this process [17,18].
BAX	0<br><br>1	BCL2:1<br><br>CASP8:1 & !BCL2	BAX represents BAX (or BAK) activation by insertion into the outer membrane of mitochondria and oligomerization.<br><br>Anti-apoptotic BH3 family members such as BCL2 inhibits BAX/BAK activation by direct interactions and/or by sequestration of pro-apoptotic BH3-only proteins [19].<br><br>CASP8-truncated Bid [20] induces the oligomerization of BAX and BAK and the formation of pores into the outer membrane of mitochondria [21].
BCL2	1	NFkB:1	BCL2 [22] and other anti-apoptotic BH3 family members such as Bfl1 [23] or BCL-xL [24] are NFkB target genes.
ROS	1	!NFkB & (MPT:1 | RIP1K:1)	RIP1 initiates caspase-independent necrosis pathway mediated by the ROS [7].<br><br>ROS are produced by NADPH oxidases activated downstream of TRADD, RIP1 and RAC1 [25].<br><br>NFkB target genes include antioxidants such as SOD2 [26] or FHC [27] that protect cell through ROS inhibition.
ATP	1	!MPT	Necrosis is accompanied by total depletion of ATP, as the result of the loss of $\Delta\psi m$ [28]
MPT	1	!BCL2 & ROS:1	By opposition to MOMP, MPT represents the permeabilization of necrotic mitochondria, as proposed by [29].<br><br>ROS production downstream of RIP1 kinase activation is involved in necrosis induction [30], and can target the permeability transition pore complex for MPT induction [29].<br><br>BCL2 over-expression inhibits MPT, probably through direct interaction with the permeabilization transition pore comple [31].
MOMP	1	BAX:1 | MPT:1	BAX/BAK pore formation at the outer membrane of mitochondria induces the release of intermembrane space resident proteins into the cytosol [21].<br><br>During TNF-induced necrotic cell death, MPT can lead to

			membranes disruption and release of intermembrane space resident proteins into the cytosol [29]
SMAC	1	MOMP	SMAC is released during MOMP in the cytosol [32].
cIAP	0<br><br>1	SMAC:1<br><br>(NFkB:1 | cIAP:1) & !SMAC	cIAP stands for cIAP1 or cIAP2<br><br>SMAC promotes the auto-ubiquitination and degradation of the cIAPs [8].<br><br>cIAPs are NFkB target genes [33].<br><br>Self-regulation on cIAP is introduced to allow cIAP:1 even if NFkB:0.
Cyt_c	1	MOMP	Cyt_c represents the release of cytochrome c from the mitochondria.<br><br>Cyt_c is localized in the mitochondrial intermembrane space and is part of the mitochondrial electron transport chain. It is released in the cytosol during apoptosis [21].
XIAP	1	!SMAC & NFkB:1	SMAC, once released in the cytosol, inactivates caspase inhibitor XIAP [32].<br><br>XIAP is a NFkB target gene [34].
apoptosome	0<br><br>1	XIAP:1<br><br>Cyt_c:1 & ATP & !XIAP	XIAP is an inhibitor of CASP9 [35].<br><br>Once released in the cytosol, Cyt_c assembles in a ATP/dATP-dependent manner with Apaf1, and pro-caspase 9 to form the apoptosome complex [36].
CASP3	0<br><br>1	XIAP:1<br><br>apoptosome & !XIAP	XIAP acts as a CASP3 inhibitor [32].<br><br>Activated CASP9 in the apoptosome complex induces proteolytic activation of executioner caspases [36].
cFLIP	1	NFkB:1	cFLIP is a target gene of NF-kB [37].
NonACD	1	!ATP	Necrosis is usually accompanied by total depletion of ATP [28].
Apoptosis	1	CASP3	Cleavage of executioner caspases substrates such as ICAM [38] or PARP1 [39] is considered as the execution phase of apoptosis.
Survival	1	NFkB:1	NFkB activation is considered the marker for survival.

SUPPLEMENTARY REFERENCES

1. Micheau O, Tschopp J (2003) Induction of TNF receptor I-mediated apoptosis via two sequential signaling complexes. Cell 114: 181-190.
2. Chinnaiyan AM, O'Rourke K, Tewari M, Dixit VM (1995) FADD, a novel death domain-containing protein, interacts with the death domain of Fas and initiates apoptosis. Cell 81: 505-512.
3. Holler N, Zaru R, Micheau O, Thome M, Attinger A, et al. (2000) Fas triggers an alternative, caspase-8-independent cell death pathway using the kinase RIP as effector molecule. Nat Immunol 1: 489-495.
4. Kreuz S, Siegmund D, Rumpf JJ, Samel D, Leverkus M, et al. (2004) NFkappaB activation by Fas is mediated through FADD, caspase-8, and RIP and is inhibited by FLIP. J Cell Biol 166: 369-380.
5. Festjens N, Vanden Berghe T, Cornelis S, Vandenabeele P (2007) RIP1, a kinase on the crossroads of a cell's decision to live or die. Cell Death Differ 14: 400-410.
6. Karin M, Lin A (2002) NF-kappaB at the crossroads of life and death. Nat Immunol 3: 221-227.
7. Leist M, Jaattela M (2001) Four deaths and a funeral: from caspases to alternative mechanisms. Nat Rev Mol Cell Biol 2: 589-598.
8. Varfolomeev E, Goncharov T, Fedorova AV, Dynek JN, Zobel K, et al. (2008) c-IAP1 and c-IAP2 are critical mediators of tumor necrosis factor alpha (TNFalpha)-induced NF-kappaB activation. J Biol Chem 283: 24295-24299.
9. Bertrand MJ, Milutinovic S, Dickson KM, Ho WC, Boudreault A, et al. (2008) cIAP1 and cIAP2 facilitate cancer cell survival by functioning as E3 ligases that promote RIP1 ubiquitination. Mol Cell 30: 689-700.
10. Kelliher MA, Grimm S, Ishida Y, Kuo F, Stanger BZ, et al. (1998) The death domain kinase RIP mediates the TNF-induced NF-kappaB signal. Immunity 8: 297-303.
11. Lee TH, Shank J, Cusson N, Kelliher MA (2004) The kinase activity of Rip1 is not required for tumor necrosis factor-alpha-induced IkappaB kinase or p38 MAP kinase activation or for the ubiquitination of Rip1 by Traf2. J Biol Chem 279: 33185-33191.
12. Karin M (2006) Nuclear factor-kappaB in cancer development and progression. Nature 441: 431-436.
13. Wajant H, Pfizenmaier K, Scheurich P (2003) Non-apoptotic Fas signaling. Cytokine Growth Factor Rev 14: 53-66.
14. Thome M, Tschopp J (2001) Regulation of lymphocyte proliferation and death by FLIP. Nat Rev Immunol 1: 50-58.
15. Medema JP, Scaffidi C, Kischkel FC, Shevchenko A, Mann M, et al. (1997) FLICE is activated by association with the CD95 death-inducing signaling complex (DISC). EMBO J 16: 2794-2804.
16. Vanden Berghe T, van Loo G, Saelens X, Van Gurp M, Brouckaert G, et al. (2004) Differential signaling to apoptotic and necrotic cell death by Fas-associated death domain protein FADD. J Biol Chem 279: 7925-7933.
17. Slee EA, Harte MT, Kluck RM, Wolf BB, Casiano CA, et al. (1999) Ordering the cytochrome c-initiated caspase cascade: hierarchical activation of caspases-2, -3, -6, -7, -8, and -10 in a caspase-9-dependent manner. J Cell Biol 144: 281-292.
18. Cowling V, Downward J (2002) Caspase-6 is the direct activator of caspase-8 in the cytochrome c-induced apoptosis pathway: absolute requirement for removal of caspase-6 prodomain. Cell Death Differ 9: 1046-1056.
19. Youle RJ, Strasser A (2008) The BCL-2 protein family: opposing activities that mediate cell death. Nat Rev Mol Cell Biol 9: 47-59.
20. Li H, Zhu H, Xu CJ, Yuan J (1998) Cleavage of BID by caspase 8 mediates the mitochondrial damage in the Fas pathway of apoptosis. Cell 94: 491-501.
21. Ow YP, Green DR, Hao Z, Mak TW (2008) Cytochrome c: functions beyond respiration. Nat Rev Mol Cell Biol 9: 532-542.
22. Catz SD, Johnson JL (2001) Transcriptional regulation of bcl-2 by nuclear factor kappa B and its significance in prostate cancer. Oncogene 20: 7342-7351.
23. Zong WX, Edelstein LC, Chen C, Bash J, Gelinas C (1999) The prosurvival Bcl-2 homolog Bfl-1/A1 is a direct transcriptional target of NF-kappaB that blocks TNFalpha-induced apoptosis. Genes Dev 13: 382-387.
24. Chen C, Edelstein LC, Gelinas C (2000) The Rel/NF-kappaB family directly activates expression of the apoptosis inhibitor Bcl-x(L). Mol Cell Biol 20: 2687-2695.

25. Vanden Berghe T, Declercq W, Vandenabeele P (2007) NADPH oxidases: new players in TNF-induced necrotic cell death. Mol Cell 26: 769-771.
26. Kamata H, Honda S, Maeda S, Chang L, Hirata H, et al. (2005) Reactive oxygen species promote TNFalpha-induced death and sustained JNK activation by inhibiting MAP kinase phosphatases. Cell 120: 649-661.
27. Pham CG, Bubici C, Zazzeroni F, Papa S, Jones J, et al. (2004) Ferritin heavy chain upregulation by NF-kappaB inhibits TNFalpha-induced apoptosis by suppressing reactive oxygen species. Cell 119: 529-542.
28. Nicotera P, Leist M, Ferrando-May E (1998) Intracellular ATP, a switch in the decision between apoptosis and necrosis. Toxicol Lett 102-103: 139-142.
29. Galluzzi L, Kroemer G (2008) Necroptosis: a specialized pathway of programmed necrosis. Cell 135: 1161-1163.
30. Morgan MJ, Kim YS, Liu ZG (2008) TNFalpha and reactive oxygen species in necrotic cell death. Cell Res 18: 343-349.
31. Kroemer G, Galluzzi L, Brenner C (2007) Mitochondrial membrane permeabilization in cell death. Physiol Rev 87: 99-163.
32. Du C, Fang M, Li Y, Li L, Wang X (2000) Smac, a mitochondrial protein that promotes cytochrome c-dependent caspase activation by eliminating IAP inhibition. Cell 102: 33-42.
33. Wang CY, Mayo MW, Korneluk RG, Goeddel DV, Baldwin AS, Jr. (1998) NF-kappaB antiapoptosis: induction of TRAF1 and TRAF2 and c-IAP1 and c-IAP2 to suppress caspase-8 activation. Science 281: 1680-1683.
34. Turner DJ, Alaish SM, Zou T, Rao JN, Wang JY, et al. (2007) Bile salts induce resistance to apoptosis through NF-kappaB-mediated XIAP expression. Ann Surg 245: 415-425.
35. Srinivasula SM, Hegde R, Saleh A, Datta P, Shiozaki E, et al. (2001) A conserved XIAP-interaction motif in caspase-9 and Smac/DIABLO regulates caspase activity and apoptosis. Nature 410: 112-116.
36. Li P, Nijhawan D, Budihardjo I, Srinivasula SM, Ahmad M, et al. (1997) Cytochrome c and dATP-dependent formation of Apaf-1/caspase-9 complex initiates an apoptotic protease cascade. Cell 91: 479-489.
37. Kreuz S, Siegmund D, Scheurich P, Wajant H (2001) NF-kappaB inducers upregulate cFLIP, a cycloheximide-sensitive inhibitor of death receptor signaling. Mol Cell Biol 21: 3964-3973.
38. Liu ZG, Hsu H, Goeddel DV, Karin M (1996) Dissection of TNF receptor 1 effector functions: JNK activation is not linked to apoptosis while NF-kappaB activation prevents cell death. Cell 87: 565-576.
39. Tewari M, Quan LT, O'Rourke K, Desnoyers S, Zeng Z, et al. (1995) Yama/CPP32 beta, a mammalian homolog of CED-3, is a CrmA-inhibitable protease that cleaves the death substrate poly(ADP-ribose) polymerase. Cell 81: 801-809.
